# The potential for bi-lateral agreements in medical tourism: A qualitative study of stakeholder perspectives from the UK and India

**DOI:** 10.1186/1744-8603-7-11

**Published:** 2011-05-03

**Authors:** Melisa Martínez Álvarez, Rupa Chanda, Richard D Smith

**Affiliations:** 1 Department of Global Health and Development, London School of Hygiene and Tropical Medicine, 15-17 Tavistock Place, London WC1H 9SH, UK; 2 Indian Institute of Management Bangalore, Bannerghatta Road, Bangalore, India

## Abstract

**Background:**

Globalisation has prompted countries to evaluate their position on trade in health services. However, this is often done from a multi-lateral, rather than a regional or bi-lateral perspective. In a previous review, we concluded that most of the issues raised could be better addressed from a bi-lateral relationship. We report here the results of a qualitative exercise to assess stakeholders' perceptions on the prospects for such a bi-lateral system, and its ability to address concerns associated with medical tourism.

**Methods:**

30 semi-structured interviews were carried out with stakeholders, 20 in India and 10 in the UK, to assess their views on the potential offered by a bi-lateral relationship on medical tourism between both countries. Issues discussed include data availability, origin of medical tourists, quality and continuity of care, regulation and litigation, barriers to medical tourism, policy changes needed, and prospects for such a bi-lateral relationship.

**Results:**

The majority of stakeholders were concerned about the quality of health services patients would receive abroad, regulation and litigation procedures, lack of continuity of care, and the effect of such trade on the healthcare available to the local population in India. However, when considering trade from a bi-lateral point of view, there was disagreement on how these issues would apply. There was further disagreement on the importance of the Diaspora and the validity of the UK's 'rule' that patients should not fly more than three hours to obtain care. Although the opinion on the prospects for an India-UK bi-lateral relationship was varied, there was no consensus on what policy changes would be needed for such a relationship to take place.

**Conclusions:**

Whilst the literature review previously carried out suggested that a bi-lateral relationship would be best-placed to address the concerns regarding medical tourism, there was scepticism from the analysis provided in this paper based on the over-riding feeling that the political 'cost' involved was likely to be the major impediment. This makes the need for better evidence even more acute, as much of the current policy process could well be based on entrenched ideological positions, rather than secure evidence of impact.

## Background

In an increasingly globalised world, countries are assessing their position on trade in health services [1]. Debates on the subject often centre on the World Trade Organisation's General Agreement on Trade in Services [2]. This is removed from the reality, however, where most trade takes place regionally or bi-laterally [3]. In addition, given that there is no systematic collection of data on the quantity of trade in health services that takes place, or the impact it has on the health system [1], discussions on whether to liberalize this type of trade tend to be more influenced by ideology than evidence [4]. Different types of evidence are needed on this debate. These include basic data on trade flows across countries, evidence of the impact of this type of trade on economies and health systems, and a greater understanding of how such trade is perceived, in terms of those involved in it, as barriers or facilitators, and the relative benefits and risks it presents. It is therefore important when seeking to understand and advise a country's position on trade to understand the various perspectives of those involved [5]. To our knowledge, such an analysis of stakeholder perspectives has not been undertaken in the area of trade and health, which this paper seeks to address.

The context for the study is *medical tourism*, as this is one of the highest profile modes of trade in health services (along with health worker migration) [1]. Medical tourism can be defined as the movement of patients across an international border for the purposes of obtaining healthcare. This is usually motivated by long waiting times, high costs of medical care or unavailability of care in the patients' home country [6,7]. This definition includes both patients paying out of pocket and the potential scenarios where governments or insurance companies pre-arrange foreign healthcare treatment for patients. Although currently the vast majority of medical tourism occurs from patients arranging and paying for the medical services abroad themselves, arguably the biggest *potential *for this market lies within governments or insurance companies pre-arranging foreign care for patients. It is therefore the potential for the development of this latter form of medical tourism that this paper concentrates upon. Currently most medical tourism takes place by individuals privately arranging healthcare in a foreign country. Often, medical tourism facilitators play a role in this by providing information on countries, hospitals and services, and sometimes even arranging the care for the patients. This, however, occurs under general regional or global agreements, which are by definition multi-lateral.

There are no agreed figures for the number of people who go abroad for healthcare, and the data available in the literature are wide-ranging (from thousands to millions) [8]. There have been several reports from consultancy firms which are often quoted to portray medical tourism as a market with great potential, but it is often unclear how their data were generated [9-11]. Medical tourism raises issues of quality of care [12], litigation [13,14], continuity of care [15], equity [16] and the creation of an internal brain drain, where medical professionals leave the public health service to work for the private hospitals that cater for (more profitable) foreign patients, further exacerbating staff shortages [17]. Countries need to take these issues into account when deciding on whether (and to what extent) to liberalize trade in health services. However, a missing piece in the discussion thus far has been a focus upon what type of trade relationship they may wish to engage in.

The literature concerning medical tourism is expanding, and there have been several recent attempts to synthesise what is already known about this practice [4,8,18-20]. Although these reviews take different perspectives on medical tourism, they all agree on the potential benefits and dangers it can bring to *importing *and *exporting *countries. Importing countries (those where the medical tourists come from, as these countries are importing healthcare services) can benefit from lower costs and reduced waiting lists; however, there are concerns regarding quality of services and litigation procedures. On the other hand, *exporting *countries (those that provide the health services to foreign patients) can bring in foreign exchange and may prevent health professionals leaving their country to work in overseas institutions, but risk creating a two-tiered system, with foreign patients receiving better care than domestic patients. In addition, reviews highlight the dearth of primary data available on medical tourism, both at the national and international level, and call for more empirical research to be carried out to ascertain the numbers of medical tourists and the procedures they undergo, as well as the validity of the claims made for and against this practice. Finally, the literature highlights the need for more and better regulatory frameworks for medical tourism, such as the creation of specific national medical guidelines to guide the provision of services to foreign patients [21], the adoption of equitable buying guidelines to address the impacts of medical tourism on equity [19], and the development of a common international regulatory platform and reporting system, that goes beyond the current regulatory bodies, such as the Joint Commission International [4]. Nonetheless, these alternative frameworks do not address the principle trade-agreement between nation states, and in this respect this paper adds to the literature by explicitly seeking to gain information relating to the potential for a bi-lateral relationship to overcome some of the core concerns raised thus far in the context of multi-lateral trade agreements (e.g. GATS).

To explore the range of issues, risks and opportunities offered to countries when engaging in bi-lateral - rather than multi-lateral - trade in more depth, the authors conducted a systematic literature review, which is reported elsewhere [Smith R, Martínez Álvarez M, Chanda R: Medical Tourism: a review of the literature and analysis of a role for bi-lateral trade, Submitted]. This literature review focussed on the UK and India as a case study of how the issues highlighted by the literature could be addressed from a bi-lateral perspective. The review supported the findings of the wider literature, as reported earlier, regarding the lack of empirical data on medical tourism and the potential benefits and risks that both importing and exporting countries face when engaging in this type of trade.

The review did, however, conclude that most of the issues that arose could be better addressed from a bi-lateral relationship, where a contract can be drawn out between the two countries outlining, for instance, which hospitals would provide the care, what procedures would be followed should something go wrong, what care the patients would receive before and after going abroad, and how this relationship could improve, rather than damage, the healthcare available to the local population. Nonetheless, there are still questions concerning the perception of such a bi-lateral system, and its ability to address concerns associated with medical tourism.

In an effort to contribute further to this literature-based evidence, the authors thus subsequently carried out semi-structured interviews with key stakeholders in both India and in the UK. The aim was to elicit their views on the prospects and impediments for a bi-lateral trade relationship in medical tourism *per se*, and specifically one between the UK and India as the principle case-study context for discussion (where the UK was seen as an importer and India as an exporter of medical services). This paper summarises the results from these interviews. Following this introduction, the methodology used to undertake and analyse the interviews is provided, followed by the results. The discussion of these results is set within the context of the results from the previous literature review, with the paper concluding with lessons for further research and policy in this area.

## Methods

A total of 30 semi-structured interviews were carried out, 20 in India and 10 in the UK. These two countries were chosen because: (i) the UK has some experience with cross-border mobility of patients within the European Union; (ii) India has already made some inroads as an exporter of health services, for instance in the provision of telemedicine services, and is one of the biggest players in medical tourism; and (iii) the two countries have historical ties, a common language, commonalities in the educational system, and there is a large Indian Diaspora population living in the UK, which would facilitate this type of trade. The balance in the number of interviews between the two countries reflects the wider range of stakeholders in India, as a destination for medical tourism, given the variety of the different providers of health services, and the different levels of government and private sector that would be involved in such a decision. The initial participants were selected to represent the key stakeholders likely to be involved in medical tourism as identified through the systematic review mentioned earlier; these were healthcare providers, members of the Department of Health and medical tourists. These stakeholders were accessed through a variety of methods. Healthcare providers and Department of Health officials were selected according to their role. Medical tourists were accessed through personal contacts of the authors. During the interviews, a snowball sampling technique was used to identify others that may have interest and be relevant to the study. The aim of using this type of sampling was to maximise the range of opinions and perspectives and to get as complete a picture on the subject as possible. Recruitment was undertaken until saturation was felt to have been reached (that no new substantive issues were being raised). The stakeholders that took part are listed in table 1. In addition, workshops were held in both India and the UK before and after the interviews took place with officials from the Department of Health, researchers, think tanks, medical professionals and hospital managers. The aim of the workshops was twofold: (i) before the interviews took place, to map out the issues and different perspectives on medical tourism, in order to guide the development of the semi-structured interview schedule; and (ii) after the interviews, to corroborate the findings and obtain any further insights.

**Table 1:** Stakeholders that took part in the study

Stakeholder group	Country	Stakeholder Number
Department of Health	UK	1, 2, 3,

Ministry of Health	India	20,21

Healthcare provider	UK	4

Healthcare provider	India	13, 14, 16, 17, 18, 19, 22, 23, 24, 25, 28,

Medical tourism facilitator^1^	UK	6

Medical tourism facilitator^1^	India	27

Think tank	UK	7

Medical tourists and companions	UK	8, 9, 10

NGO	India	11

Academic	India	26

Insurance company	India	29,30

Industry association	UK	5

Industry association	India	12, 15

^1^In the category of Medical tourism facilitators we included agencies that either pre-arranged services for the medical tourists (including transport, hospital stay, medical procedures, accompanying person) or provided information services on medical tourism, destinations and providers to potential medical tourists.

The interviews took place between November 2009 and January 2010 and were carried out by one of the authors in the UK (MMA) and another author in India (RC). The interviews lasted approximately one hour, and were semi-structured in nature [22]. This required a qualitative instrument to be designed to guide the interviews; as indicated, this was based on material obtained from the literature review and the first workshops [Smith R, Martínez Álvarez M, Chanda R: Medical Tourism: a review of the literature and analysis of a role for bi-lateral trade, Submitted]. An example is presented in Additional file 1: Appendix A. The instrument was used as a topic guide, but not all questions were covered in all of the interviews, nor were they asked in strictly the order that they are presented. Rather, the instrument was adapted to each participant. The broad themes were: general perspectives, medical tourism-related activities and operations, and policy issues.

The interviews were conducted face-to-face or over the telephone, and the answers recorded by hand, and then typed up. Written consent was obtained from each interviewee before the interview took place.

The interview transcripts were analysed using thematic [23] and directed content analysis [24]. The scripts were reviewed and themes were identified. These were coded based on the results from the literature review and the workshops carried out previously by the authors. Any additional themes that arose from the transcripts were incorporated (see table 2 for a complete list of coding categories). This was carried out by the first author (MMA), and approved by the other authors (RC and RS). No software was used in the analysis of the interviews.

**Table 2:** Coding categories and frequency by groups

Coding Category^1^	Frequency by groups										
	**Government officials**		**Healthcare providers**		**Medical Tourism Facilitators**		**Medical Tourists (UK nationals)**	**Industry associations**		**Insurance company**	**Others^1^**

	**UK**	**India**	**UK**	**India**	**UK**	**India**		**UK**	**India**		

Data				8	1	1					1

Countries		1		10		1			2		2

Regulation	1			8		1			1	1	2

Quality		1	1	4		1	1		1	1	3

Litigation	2		1	2		1	2		2		1

three hour "rule"	2	1		3			2		2		2

Role of government	1			6		1			1		4

Diaspora	2	2	1	6			1		1		3

Local population	1	1		3		1			1		3

Continuity of care	1		1	1			2				1

Prospects	2		1	2	1						1

Perception of India			3		1			1		1	1

^1^The coding categories were identified from the interview transcripts, and influenced by the literature search. They are summarised here. **Data: **Any data that the participant had available on the size of the medical tourism market, either in global/national terms or numbers from their institution. **Countries: **Participants were asked to name countries involved in medical tourism, both as importers and exporters. **Regulation: **Although not specifically asked for, the lack of regulation of the medical tourism market, and the perceived lack of regulation in health services in the exporting country were mentioned by a significant number of the participants as a key concern. **Quality: **Similarly, the quality of the healthcare services obtained abroad was a key concern for the stakeholders interviewed. **Litigation: **Many stakeholders showed concerns about the differences in malpractice laws and litigation procedures between the exporting and importing countries. **Three hour 'rule': **One of the key barriers highlighted specific to the UK market, the three hour flight restriction on how far patients can travel to obtain healthcare was also highlighted by the respondents. **Role of government: **A bi-lateral agreement between the UK and India on medical tourism would need both governments to be involved; we therefore asked participants on what role (if any) they thought government should have in this. **Diaspora: **The Diaspora appeared to be a key target audience for medical tourism, as the perception was that many would prefer to return "home" for care. **Local population: **Another key concern highlighted in the literature and many of the interviews was the impact medical tourism would have on the healthcare available to the local population. **Continuity of care: **Many of the stakeholders highlighted the lack of continuity of care a patient would experience when going abroad as a major issue. Additionally, some also feared they may cause a bigger burden on the health system if they have complications once they return. **Prospects: **This category included respondents' views on the prospects for a bi-lateral trade relationship on medical tourism between the UK and India. **Perception of India: **Participants' perception of India was a key influence on their views on the viability for this type of trade relationship between the two countries.

Think tank, NGO, Public Health Foundation of India

## Results

A summary of all the coding categories and the frequency of these by the different groups of stakeholders are shown in table 2. The core findings from this are explored in more detail below.

### Data availability

The review of the literature carried out previously by the authors had shown that there was very little data, and of poor quality, available on medical tourism flows. This is of concern, since in order to determine the scale and impact of medical tourism, data is needed by policy-makers on, for instance, the numbers of medical tourists, the revenues these bring, the outcomes of treatment and the impacts medical tourism has on the home and destination health system. Some of the Indian healthcare providers interviewed estimated that foreign patients made up 5-7% of all patients they treated, with foreign patients representing a slightly higher share of revenue source (although this figure includes foreign patients that were not medical tourists). Many respondents quoted figures from reports carried out by the Confederation of Indian Industries, McKenzie and Deloitte suggesting the promising *prospects *for medical tourism, rather than actual current figures, whilst others, such as medical tourism facilitators, were highly critical of these reports:

*Medical tourism is a much overhyped market. People have a vested interest saying it is the next big thing, but the numbers are exaggerated ... However, because of these high figures, people see it as a growth market, which results in too many organisations in the market (hospitals, countries, agents) but not enough patients to go round*. (Respondent #6, UK medical tourism facilitator)

In general, it was clear that the stakeholders interviewed had very little information on the current status of medical tourism in general, and that related to India and the UK specifically, generating concern for the development of policy concerning this area.

### Origin of medical tourists

Most respondents commented on the target population for the private hospitals in India that attract medical tourists. When asked about which countries international patients came from, most participants from India named neighbouring and low-income countries, with the UK rating low on their priority list. A typical response was that:

*The bulk of foreign patients come from Afghanistan, Bangladesh, Nigeria, Iraq, and Ethiopia. The SAARC *[South Asian Association for Regional Coordination] *countries account for 50% of all foreign patients, another 20-25% are from Afghanistan, 3.5% or so from the Middle East, 3% from the US ... The UK is not a large market *(Respondent #18, Indian healthcare provider)

There was also widespread agreement, both from the respondents in the UK and in India, that the Diaspora community would be willing to travel back "home" for treatment. However, opinion was divided on the significance of this population. Most of the UK and some of the Indian stakeholders believed that the number of these potential travellers would be small in absolute terms:

*There are small numbers of citizens of an Indian origin that would benefit from this type of arrangement, for cultural or religious reasons, but the numbers are small *(Respondent #1, UK Department of Health)

Whereas, the majority of the Indian participants believed them to be a key target group for medical tourism:

*The main audience could be the NRI *[Non-Resident Indian] *population in these developed countries *(Respondent #12, Indian industry association);

Nonetheless, there was widespread agreement between stakeholders that hospitals in India should concentrate on the large domestic market, before targeting foreign patients; this was the view of government officials in both countries, members of academic and industry associations, although, perhaps predictably, only a small proportion of the Indian healthcare providers.

*Indian private hospitals argue they have a lot of extra capacity. However, it is hard to believe they are covering the whole of the Indian population *(Respondent #5, UK industry association)

*The negative part of this is that to chase that foreign segment of patients, we may end up neglecting others needing healthcare in our own country *(Respondent #11, Indian NGO)

The medical tourists interviewed did not comment on this issue.

### Quality, perception, regulation and litigation

The majority of the UK stakeholders interviewed showed concerns regarding the quality of healthcare in India, and a potential lack of regulation.

*In the UK we have the Care Quality Commission, guidelines, etc., but how do you ensure that the standards are the same or better? *(Respondent #4, UK healthcare provider)

However, this was contradicted by most of their Indian counterparts, who believed healthcare providers in India have made great progress in obtaining national and international accreditation, and that the standard of care was very high. They were, though, very aware of the poor perception that the UK had of Indian healthcare.

*The main barrier is perception ... but the procedures and staff are of top quality *... *Quality is not a problem for Indian corporate hospitals as 70-80% of their doctors are often trained in the UK and the standards are equivalent to those in the US or the UK *(Respondent #17, Indian healthcare provider)

*Now NABH *[National Accreditation Board for Hospitals & Healthcare providers] *is ISQua *[International Society for Quality in healthcare] *accredited*. *By this certification, the basic level of hospital quality is now ensured in India*. (Respondent #14, Indian healthcare provider)

Nevertheless, this was not the opinion of all Indian stakeholders: whilst most healthcare providers agreed that standards were high and regulation was necessary, some members of academic and industry associations believed the accreditation industry was not as reliable as some believe it to be, and many establishments were wasting their money getting different types of accreditation.

*There is concern that now with the NABH and the insistence on it being mandatory, quality may come down over time ... There is also corruption among the auditors and the process of accreditation can be questioned *(Respondent #22, Indian healthcare provider)

When discussing quality concerns, most participants linked these to the issue of litigation, which was a concern across the board for Indian and UK stakeholders.

*Under a bilateral agreement, care in India would be commissioned by the NHS, and therefore the NHS is automatically responsible for any litigation payments, which would outweigh the savings made *(Respondent #1, UK Department of Health)

*If one patient sues us, we can have huge problems ... We do not follow systems but have money and influence ... Our legal framework is poor. In the consumer court, the number of pending cases is huge and corruption is also at a high level *(Respondent #12, India industry association)

*There are concerns about medical malpractice. The company has decided to stay away from issues of liability. There are indemnity clauses built in *(Respondent #27, Indian medical tourism facilitator)

Interestingly, the medical tourists that took part in the interviews did not think they had the right to any sort of compensation, only one had ensured that the hospital would take full responsibility and carry out any further procedures for free should anything have gone wrong. One medical tourist had experienced bad quality and potential medical malpractice, but no action was taken.

*Many weeks after the surgery she *[respondent's mother] *was no better but they *[the doctors in India] *did not listen to us and told us that it would get better in time. My mum's problem has got worse with more complications and the doctors here said the surgery there was not required. We believe that the doctor there used us just to make a profit *(Respondent #10, medical tourist companion).

### Three hour 'rule'

One of the most controversial issues was the three-hour 'rule'. This 'rule' was supposedly devised by the UK Department of Health when considering sending patients for care to other EU countries, as part of EU-wide legislation, stating that patients were not allowed to be treated in countries that were more than three-hours flying time away. The participants from India thought that this was a major formal barrier for the NHS when considering sending patients to India.

*The main problem is the three hour restriction by the NHS. This has to be taken up in a bilateral dialogue with the UK on a regular basis and the time has to be increased to eight hours to cover India *(Respondent #21, Indian Ministry of Health)

However, the opinion in the UK was divided. Although respondents from industry considered it an important barrier, it was interesting that participants from both the NHS and the Department of Health considered it only "informal guidance":

*This is neither a legal rule nor formal guidance. How far someone can travel depends on the clinical needs of the individual. The three hour rule is not a formal binding, and it is not used ... it is up to consultants and PCTs *[Primary Care Trusts] *to decide how long someone can travel ... It wouldn't make sense, as there are areas within the EU that are further than 3 hours! *(Respondent #1, UK Department of Health)

The patients interviewed strongly disagreed with the 'rule':

*It's a ridiculous rule. If they are willing to consider sending patients to the EU on a case-by-case basis, then they should use that same basis to let them go further. This would not be the case for patients that would need heart surgery, but for things like knee, joints, fingers, eyes*, *etc*. (Respondent #8, medical tourist)

Clearly the status of such a 'rule', illustrative of other perceived barriers in other contexts, is of major significance to establish in any discussions about more formal trade agreements in this area.

### Continuity of care

The problem of continuity of care was highlighted by all groups of participants, both in the UK and in India. In fact, for the UK respondents, it appeared to be a more important issue than the 'three-hour rule'.

*If they *[medical tourists] *need continuity of care, especially if problems occur, who is going to be there to fix them? There would be an unwillingness here *[UK] (Respondent #4, UK healthcare provider)

Patients also showed concerns about continuity of care, although they did not express any problems in getting care once they returned.

*They *[domestic healthcare providers] *should at least arrange the aftercare and pre-treatment for those that choose to go privately, as they would be saving money by not having to operate on them *(Respondent #9, medical tourist)

### Prospects and policy changes needed

In general, respondents believed the prospects for a bi-lateral relationship in this type of trade in health services between India and the UK to be low given the present circumstances.

*The UK government ... does not see what value there would be from setting such an arrangement *(Respondent #1, UK Department of Health)

*We are not a good option for the UK *(Respondent #12, India industry association)

When asked about what policy changes would be needed to enable this relationship and what (if any) role the governments on each country should play, there was widespread disagreement amongst the participants. Although most of the UK participants showed some concern regarding the level at which changes should take place, they all believed whatever changes were required simply would not happen.

*It is never going to happen ... It could not happen politically. It may make logical sense, but it would never happen in the real world *(Respondent #6, UK medical tourism facilitator)

*A very sophisticated bilateral agreement would need to be produced. But the NHS is facing bigger problems at the moment, and no government would devote resources to achieving that* (Respondent #7, UK think tank)

The Indian participants disagreed on whether the Indian government should be actively involved in facilitating medical tourism. The majority of the respondents, including those from Indian healthcare establishments, believed that the government should be involved, whereas the respondents from the Ministry of Health and academic and industry associations did not.

*The government of India should focus on medical value travel as a source of foreign exchange. It can also help improve standards, skills, enable international accreditation and in the process help domestic patients *(Respondent #18, Indian healthcare provider)

*The official position can never be to focus on medical tourism as this would be perceived as coming at the cost of public health *(Respondent #21, Indian Ministry of Health)

Overall, although there was some indication that movement toward a bi-lateral agreement on medical tourism may make some sense on both sides, the political 'cost' involved was likely to be the major impediment to any movement in practice and in the foreseeable future.

## Discussion

Of the key themes identified, there was agreement between the stakeholders on the importance of regional travel and proximity, quality, regulation, litigation, continuity of care and the low prospects for a bilateral relationship between India and the UK on medical tourism. However, there were disagreements on the importance of the Diaspora in medical tourism, whether countries like India should concentrate on the local population before offering services to foreign patients or if they should focus on this industry to raise foreign exchange, the status of the three hour 'rule', and what policy changes would be needed for such a relationship to prosper (including the role of government).

These results are subject to two important caveats. First, two different interviewers were involved (one in the UK and one in India), and although the same interview instrument was used there may have been differences in the way questions were asked or answers recorded. Second, the stakeholders approached in India were more willing to participate in the interviews than those approached in the UK. This means that the opinion of those who were not willing to participate from the UK was not represented.

This research supports some of the concerns found in the literature, including quality of care [14], litigation [13,25], continuity of care [15,26] and the potentially harmful impact medical tourism may have on the local population [19,21,27]. However, as highlighted above, not all stakeholders were in support of these arguments. In addition, this research has shed some light on other issues, such as the uncertainty surrounding three hour 'rule', the importance of the Diaspora and has provided some indication on the amount of foreign patients that Indian private hospitals provide medical treatment to. Although the UK and India were selected as a case study in this research, this is not an exclusive example where this type of trade could take place. The UK has cultural links with many other countries where English is widely spoken that offer medical tourism services, and India attracts medical tourists from across the world.

Nevertheless, when policy makers have to make decisions on whether to engage in this type of trade, this takes place largely in a data vacuum, given that there is no data on the amount of medical tourism that goes on, and what impacts it has on the exporting and importing health system. As this research shows, opinion amongst stakeholders in this field is often divided. These issues cannot be resolved until more and better quality data becomes available.

More research is needed to determine the flows of medical tourism, including the total numbers, what countries they come from and where they get treated. Additionally, it is essential that there is more evidence on the impact medical tourism is having, both on importing and exporting countries, as at present, it is impossible to know whether the concerns outlined in the literature and in these interviews are likely to be realised. Finally, it is of particular importance to examine the effect different types of trade relationships might have. In this respect, whilst the literature review previously carried out by authors suggested that a bi-lateral relationship would be best-placed to address the concerns raised regarding medical tourism, there was scepticism from the analysis provided in this paper based on the over-riding feeling that the political 'cost' involved was likely to be the major impediment. This makes the need for better evidence even more acute, as much of the current policy process could well be based on entrenched ideological positions, rather than secure evidence of impact.

This research can also provide some policy recommendations. The literature suggests that medical tourism can cut costs, improve waiting lists (from the perspective of importing countries) and generate revenue and improve the national healthcare (from the perspective of exporting countries). Given these, policymakers should consider medical tourism as an option to improve their health services. This is particularly important in the current climate where countries are facing budget cuts spurned on by the financial crisis. When evaluating the different options available, policymakers should consider engaging in a bi-lateral relationship, as it has the most potential to ensure the interests of the patients on both countries are prioritised. This could also be addressed through the ongoing EU-India trade negotiations, which also cover services.

In conclusion, there are a wide variety of barriers affecting medical tourism, including restrictions on travel by governments and concerns about quality, regulations and litigation. In order to increase trade, these barriers need to be addressed. The results from the literature review reported elsewhere [Smith R, Martínez Álvarez M, Chanda R: Medical Tourism: a review of the literature and analysis of a role for bi-lateral trade, Submitted] suggests that a bi-lateral relationship is best-placed to tackle these barriers. However, results from the analysis reported here suggest that it is the political sensibility that is the major barrier to be overcome. In this respect, it is worth remembering that policy decisions affecting this type of trade in health services are largely taking place in a data vacuum. It is therefore imperative for more studies and surveys to be carried out and for more empirical data to be collected, in particular data on the size of the medical tourism market, pricing of services, regional breakdown of markets, and the impact of different forms of trade agreement.

## Competing interests

The authors declare that they have no competing interests.

## Authors' contributions

MMA contributed to the development of the survey instrument, carried out some of the interviews and drafted the initial paper and agreed on final version

RC conceptualised the work, contributed to the development of the survey instrument, carried out some of the interviews and reviewed, commented and agreed on final version

RS conceptualised the work, contributed to the development of the survey instrument, reviewed, commented and agreed on final version

## Supplementary Material

Additional file 1 **Appendix A. Discussion Guide for Medical Tourism Importer**. A sample discussion guide used in carrying out the interviews. Click here for file

## References

[B1] Smith RD Chanda R Tangcharoensathien V Trade in health-related services Lancet 2009 373 9663 593 601 10.1016/S0140-6736(08)61778-X 19167053

[B2] Blouin C Drager N Smith R International Trade in Health Services and the GATS: Current Issues and Debates 2005 Washington, D.C.: World Bank

[B3] Blouin C Drager N Smith R Building a national health strategy for trade: A guide for policy making Montreal: McGill University Press in press

[B4] Lunt N Carrera P Medical tourism: assessing the evidence on treatment abroad Maturitas 2010 66 1 27 32 10.1016/j.maturitas.2010.01.017 20185254

[B5] Smith RD Lee K Drager N Trade and health: an agenda for action Lancet 2009 373 9665 768 773 10.1016/S0140-6736(08)61780-8 19167056 PMC2726935

[B6] Mattoo A Rathindran R How health insurance inhibits trade in health care Health Aff (Millwood) 2006 25 2 358 368 10.1377/hlthaff.25.2.358 16522578

[B7] Carrera P Bridges J Globalization and healthcare: understanding health and medical tourism Expert Review Pharmacoeconomics Outcomes Research 2006 6 4 447 454 10.1586/14737167.6.4.447 20528514

[B8] Crooks VA Kingsbury P Snyder J Johnston R What is known about the patient's experience of medical tourism? A scoping review Bmc Health Serv Res 2010 10 266 10.1186/1472-6963-10-266 20825667 PMC2944273

[B9] Confederation of Indian Industries and McKinsey & Co Healthcare in India: The road ahead 2002 New Delhi: CII & McKinsey & Company

[B10] Deloitte Center for Health Solutions Medical Tourism: Consumers in search of value 2008 Washington, D.C.: Deloitte

[B11] Tourism Research And Marketing Medical tourism: a global analysis 2006 Arnhem: ATLAS

[B12] Canales MT Kasiske BL Rosenberg ME Transplant tourism: Outcomes of United States residents who undergo kidney transplantation overseas Transplantation 2006 82 12 1658 1661 10.1097/01.tp.0000250763.52186.df 17198255

[B13] Chinai R Goswami R Medical visas mark growth of Indian medical tourism Bull World Health Organ 2007 85 3 164 165 10.2471/BLT.07.010307 17486202 PMC2636228

[B14] MacReady N Developing countries court medical tourists Lancet 2007 369 9576 1849 1850 10.1016/S0140-6736(07)60833-2 17549816

[B15] Connell J Woodside A, Martin D Tummy tucks and the Taj Mahal? Medical tourism and the globalization of health care Tourism Management 2008 CAB International 232 244

[B16] Sen Gupta A Medical tourism in India: winners and losers Indian J Med Ethics 2008 5 1 4 5 18630244 10.20529/IJME.2008.002

[B17] Arunanondchai J Fink C Trade in health services in the ASEAN region Health Promot Int 2006 21 Suppl 1 59 66 17307958 10.1093/heapro/dal052

[B18] Hopkins L Labonte R Runnels V Packer C Medical tourism today: what is the state of existing knowledge? J Public Health Policy 2010 31 2 185 198 10.1057/jphp.2010.10 20535101

[B19] Johnston R Crooks VA Snyder J Kingsbury P What is known about the effects of medical tourism in destination and departure countries? A scoping review Int J Equity Health 2010 9 24 10.1186/1475-9276-9-24 21047433 PMC2987953

[B20] Reddy S Qadeer I Medical tourism in India: Progress or predicament? Economic and Political Weekly 2010 45 20 69 75

[B21] Shetty P Medical tourism booms in India, but at what cost? Lancet 2010 376 9742 671 672 10.1016/S0140-6736(10)61320-7 20879077

[B22] Coast J The appropriate uses of qualitative methods in health economics Health Econ 1999 8 4 345 353 10.1002/(SICI)1099-1050(199906)8:4<345::AID-HEC432>3.0.CO;2-Q 10398527

[B23] Braun V Clarke V Using thematic analysis in psychology Qualitative Research in Psychology 2006 3 77 101

[B24] Hsieh HF Shannon SE Three approaches to qualitative content analysis Qual Health Res 2005 15 9 1277 1288 10.1177/1049732305276687 16204405

[B25] Turner L "First world health care at third world prices": globalization, bioethics and medical tourism BioSocieties 2007 2 303 325 10.1017/S1745855207005765

[B26] Cheung IK Wilson A Arthroplasty tourism Med J Aust 2007 187 11 12 10.5694/j.1326-5377.2007.tb01467.x 18072912

[B27] Garud AD Medical tourism and its impact on our healthcare Natl Med J India 2005 18 6 318 319 16483034

