# Supplementary material for: The potential for bi-lateral agreements in medical tourism: A qualitative study of stakeholder perspectives from the UK and India
Source: Global Health. 2011 May 3;7:11. doi: 10.1186/1744-8603-7-11 (PMC3110115; doi:10.1186/1744-8603-7-11)
Supplement: Additional file 1 — Appendix A. Discussion Guide for Medical Tourism Importer. A sample discussion guide used in carrying out the interviews. [file 1744-8603-7-11-S1.DOCX]

Appendix A

**Discussion Guide for Medial Tourism Importer**

**Research Project on**

**Prospects for Telemedicine and Medical Tourism between India and the UK**

**I. Background information**

Name: _______________________________ Designation: _______________________________

Name of organization: _______________________________

Location: _______________________________

Date and time: _______________________________

Contact address

__________________________________________________________________________________________________________________________________________________________

Contact phone number/s:_________________________________

Email contact: _________________________________

Brief profile of organization: __________________________________________________________________________________________________________________________________________________________

Size: ___________________________

**II. General Perspectives**

1. What are your views on medical tourism? Do you think there is potential for this trade? How much do you know about this type of trade?

____________________________________________________________________________________________________________________________________________________________________________________________________________________________________________________________________________________________________________________________________________________

1. How promising do you think the prospects are for medical tourism trade at the global level?

Very high ____ Moderate ________ Low ________

Why?

________________________________________________________________________________________________________________________________________________________________________

1. Which are the main regions and countries where you see prospects for such trade globally? List up to 5 in each case.

| Top 5 regions/  countries | Exporting regions | Exporting countries | Importing regions | Importing countries |
| --- | --- | --- | --- | --- |
| 1 |  |  |  |  |
| 2 |  |  |  |  |
| 3 |  |  |  |  |
| 4 |  |  |  |  |
| 5 |  |  |  |  |

1. Which are the main medical tourism sectors where you see prospects globally? (e.g. cataract) List up to 10.

________________________________________________________________________________________________________________________________________________________________________

1. How promising do you find the prospects for medical tourism exports from India?

Very high ____ Moderate ________ Low ________

Why?

________________________________________________________________________________________________________________________________________________________________________

1. In your view, is the demand for medical tourism more likely to come from the private or from the public sector?

_____________________________________________________________________________

_____________________________________________________________________________

1. Are you aware of any barriers to medical tourism?

_____________________________________________________________________________

_____________________________________________________________________________

1. Are you aware of the discussions on trade in health services under the General Agreement on Trade in Services in the World Trade Organisation? Yes _______ No _______
2. What impact do you think medical tourism has on the host population?

____________________________________________________________________________

____________________________________________________________________________

And on the host health system?

_____________________________________________________________________________

____________________________________________________________________________________

1. What impact do you think medical tourism has on the population of the *importing* country?

______________________________________________________________________________

____________________________________________________________________________________

And on its health system?

_____________________________________________________________________________

_____________________________________________________________________________

1. Are there any disadvantages to medical tourism? Yes _______ No _______

If yes, which ones? ________________________________________________________________________________________________________________________________________________________

1. What is the evidence base for the views expressed above, both generally and with specific regard to India and the UK?

Yes No

Secondary sources (newspaper and magazine reports, articles) _____ ______

Interactions with government organizations _____ ______

Interactions with industry players _____ ______

Conferences/seminars/meetings _____ ______

Operations and activities of your current/earlier organization/s _____ ______

Other (please state) __________________________________________________________

15. Are there any specific data sources you would recommend for information on medical tourism?

______________________________________________________________________________

**III. Activities and Operations**

1. Does your organization (DoH, NHS, PCT, Hosp) send patients abroad for treatment? Yes ____ No ____

2. If so, please list the top 5 countries to which you sent patients in each of the past three years and the associated numbers.

|  | 2006 | | 2007 | | 2008 | |
| --- | --- | --- | --- | --- | --- | --- |
| Sl No. | Country | No. or Share of patients sent abroad a/ | Country | No. or share  of foreign patients a/ | Country | No. or share  of foreign patients a/ |
| 1 |  |  |  |  |  |  |
| 2 |  |  |  |  |  |  |
| 3 |  |  |  |  |  |  |
| 4 |  |  |  |  |  |  |
| 5 |  |  |  |  |  |  |

a/ Note: If you are unable to provide absolute numbers, provide the share of patients sent abroad accounted for by each country.

1. What are the main treatments that patients are sent abroad for? Tick all that are applicable below.

Cardiac surgery ________

Hip replacement ________

Cataract surgery ________

Cosmetic surgery ________

Dental treatment ________

Joint surgery ________

IVF treatment ________

Organ transplants ________

Other ________

If you ticked “Other”, please list up to 5 other treatments _____________________________________________________________________________________

1. What are the main reasons why foreign patients go abroad for treatment?

**____________________________________________________________________________**

**____________________________________________________________________________**

1. What are your main concerns in sending patients to countries like India for treatment?

**____________________________________________________________________________**

**____________________________________________________________________________**

1. In your view, which are the competing destinations to India?

**_____________________________________________________________________________**

**_____________________________________________________________________________**

1. What are the main benefits the UK can derive from medical tourism?

**____________________________________________________________________________**

**______________________________________________________________________________**

1. What are your views on the current 3-hour flight restriction for sending patients abroad?

**____________________________________________________________________________**

**____________________________________________________________________________**

1. What are your views on the prospect of sending patients to get treatment in India?

**_____________________________________________________________________________**

**_____________________________________________________________________________**

1. Do you think such overseas medical treatment should be sponsored by the NHS or should it remain private, out of pocket only? Why?

**_____________________________________________________________________________**

**_____________________________________________________________________________**

**_________________________________________________________________________________**

1. Would you prefer sending patients to other parts of Europe (or EU) as opposed to India? Why?

**_____________________________________________________________________________**

**______________________________________________________________________________**

1. Could you comment on recent reforms and policy initiatives in healthcare in the UK, which would have a bearing on the prospects for medical tourism imports by the UK? Please list some of these specific measures.

**_____________________________________________________________________________**

**_____________________________________________________________________________**

**_____________________________________________________________________________**

**_____________________________________________________________________________**

**IV. Policy Issues**

1. How can medical tourism exports to the UK be increased? List a few specific steps that need to be taken by the governments on both sides.

In the UK In India

_______________________________________ ____________________________________

_______________________________________ ____________________________________

_______________________________________ ____________________________________

1. List up to 3 specific steps the industry could take in India to increase medical tourism exports to the UK.

______________________________________________

______________________________________________

______________________________________________

1. How optimistic are you about enhancing this bilateral relationship in medical tourism trade?

Very optimistic ______________ Moderately optimistic ______________ Not at all optimistic ______

Why?

_____________________________________________________________________________

_____________________________________________________________________________

1. If you are moderately optimistic, in what kind of timeframe do you expect improved prospects?

______________________________________________________________________________

1. Do you think partnerships and tie-ups or pilot arrangements could enhance medical tourism exports from India to the UK?

Yes ___________ No ___________

1. Do you think the India-EU Trade and Investment Agreement that is currently under negotiation could enhance prospects for medical tourism trade between the two countries?

Yes ____________ No ____________

List up to 3 specific issues that would need to be addressed under this agreement to enhance prospects for medical tourism exports from India to the UK?

_____________________________________________________________

_____________________________________________________________

_____________________________________________________________

1. Please list any other issues you would like to highlight and which have not been raised above.

_______________________________________________________________________

____________________________________________________________________________________

**Thank you**
